# Glycoproteomic Characterization of FUT8 Knock-Out CHO Cells Reveals Roles of FUT8 in the Glycosylation

**DOI:** 10.3389/fchem.2021.755238

**Published:** 2021-10-29

**Authors:** Ganglong Yang, Qiong Wang, Lijun Chen, Michael J. Betenbaugh, Hui Zhang

**Affiliations:** ^1^ Department of Pathology, Johns Hopkins University, Baltimore, MD, United States; ^2^ Department of Chemical and Biomolecular Engineering, Johns Hopkins University, Baltimore, MD, United States

**Keywords:** glycoproteomics, FUT8 knock-out CHO cells, mass spectrometry, intact glycopeptides, glycosylation related enzyme

## Abstract

The α1,6-fucosyltransferase (encoded by FUT8 gene) is the key enzyme transferring fucose to the innermost GlcNAc residue on an N-glycan through an α-1,6 linkage in the mammalian cells. The presence of core fucose on antibody Fc region can inhibit antibody-dependent cellular cytotoxicity (ADCC) and reduce antibody therapeutic efficiency *in vivo*. Chinese hamster ovary (CHO) cells are the predominant production platform in biopharmaceutical manufacturing. Therefore, the generation of FUT8 knock-out (FUT8KO) CHO cell line is favorable and can be applied to produce completely non-fucosylated antibodies. The characterization of monoclonal antibodies as well as host cell glycoprotein impurities are required for quality control purposes under regulation rules. To understand the role of FUT8 in the glycosylation of CHO cells, we generated a FUT8 knock-out CHO cell line and performed a large-scale glycoproteomics to characterize the FUT8KO and wild-type (WT) CHO cells. The glycopeptides were enriched by hydrophilic chromatography and fractionated 25 fractions by bRPLC followed by analysis using high-resolution liquid chromatography mass spectrometry (LC-MS). A total of 7,127 unique N-linked glycosite-containing intact glycopeptides (IGPs), 928 glycosites, and 442 glycoproteins were identified from FUT8KO and WT CHO cells. Moreover, 28.62% in 442 identified glycoproteins and 26.69% in 928 identified glycosites were significantly changed in the FUT8KO CHO compared to wild-type CHO cells. The relative abundance of all the three N-glycan types (high-mannose, hybrid, and complex) was determined in FUT8KO comparing to wild-type CHO cells. Furthermore, a decrease in fucosylation content was observed in FUT8KO cells, in which core-fucosylated glycans almost disappeared as an effect of FUT8 gene knockout. Meantime, a total of 51 glycosylation-related enzymes were also quantified in these two cell types and 16 of them were significantly altered in the FUT8KO cells, in which sialyltransferases and glucosyltransferases were sharply decreased. These glycoproteomic results revealed that the knock-out of FUT8 not only influenced the core-fucosylation of proteins but also altered other glycosylation synthesis processes and changed the relative abundance of protein glycosylation.

## 1 Introduction

Chinese hamster ovary (CHO) cells have been extensively applied in pharmaceutical manufacturing to produce therapeutic recombinant proteins, especially monoclonal antibodies. The merits of CHO cell culture include the adaption ability to grow at high densities in suspension cultures, high levels of protein expression over prolonged fermentation cycles, the incorporation of complex glycans on exogenously expressed proteins and the capacity to perform post-translational modifications compatible with humans (Byrne et al., 2018; Mizukami et al., 2018). Currently, CHO cells are involved in the production of more than 70% of recombinant biopharmaceutical proteins, most of them being monoclonal antibodies (mAbs) (Lalonde and Durocher, 2017). The glycan profiles on recombinant glycoproteins, including monoclonal antibodies, have shown different influences on their biological, pharmacokinetic, and immunogenic properties (Sethuraman and Stadheim, 2006). The diversity and complexity of these N-glycans can be attributed to the high number of different sugar moieties and the multitude of possible linkages. For example, mannosylation affects pharmacokinetics (Liu, 2015). Sialylation is associated with anti-inflammatory properties, and terminal galactosylation involves in complement dependent cytotoxicity (Peschke et al., 2017; Aoyama et al., 2019; Markina et al., 2020). Core fucosylation impacts protein activity, antibody-dependent cell-mediated cytotoxicity (ADCC) and antibody-dependent cell-mediated phagocytosis (Chang et al., 2019). Therefore, the glycopatterns on therapeutic proteins are an important quality attribute and the control and monitoring of glycosylation is necessary for the pharmaceutical industry to maintain lot-to-lot consistency.

Given its non-template-driven nature, various parameters, intracellularly and extracellularly, can affect the final glycoforms on glycoproteins in mammalian cells. There are two strategies to control the glycosylation of therapeutic proteins, manipulation of culture conditions and gene editing. Process factors such as pH, ammonia level, dissolved O_2_, temperature and bioreactor mode can influence the glycosylation in CHO cells (Wong et al., 2010). Precise gene editing provides a nearly unlimited playground for producing tailored glycans on therapeutic proteins. Moreover, gene editing of glycosylation in mammalian cells could contribute tremendously to the current view of biosynthetic regulation of the cellular glycome and highlight important functions of glycans involved in development, health, and disease (Byrne et al., 2018; Narimatsu et al., 2021). Several glycoengineering strategies have emerged to recreate these beneficial profiles on recombinant proteins. Afucosylation presents one of the most popular glycoengineering approaches for biologics (Lalonde and Durocher, 2017). Afucosylation has been achieved by knocking out FUT8 or the other enzymes involved in the biosynthesis of guanine diphosphate-fucose (Cristea et al., 2013; Sun et al., 2015).

The genomics, transcriptomics, proteomics, metabolomics, glycomics and other omics were extensively applied in the therapeutic protein bioproduction of CHO cells to maximize gains from CHO engineering and bioprocess improvements. Glyco-analytical technologies are essential for the characterization of therapeutic glycoproteins for drug discovery, development, and for quality control of lot and batch variations to ensure safety and efficacy of the biotherapeutic (O'Flaherty et al., 2018). Glycoproteomics is a powerful tool for glycoprotein analysis, which could identify the diverse glycan sites on each glycoprotein as well as characterize the heterogeneous glycoforms at each site (Kolarich et al., 2012; Gutierrez Reyes et al., 2021). To understand the role of FUT8 in the glycosylation of CHO cells, we analyzed the glycoproteome of FUT8KO and wild-type CHO-K1 cells (Sun et al., 2016; Yang et al., 2018). We enriched the glycopeptides by hydrophilic column MAX and fractionated 25 fractions by basic reversed-phase liquid chromatography (bRPLC). The intact glycopeptides were analyzed by high-resolution LC-MS/MS and annotated by GPQuest. From our results, it revealed that the FUT8KO not only influenced the glycosylation of proteins but also changed the relative abundance of some proteins.

## 2 Materials and Methods

### 2.1 1FUT8 Knock-Out CHO Cell Culture and Protein Digestion

To silence α-1,6-fucosylation on antibodies expressed in CHO cells, we applied CRISPR-Cas9 method to disrupt the α-1,6-fucosyltranferase (FUT8) gene (Wang et al., 2018). The stable FUT8KO and wild-type (WT) CHO cells were adapted to suspension cell culture status in CD-CHO media with 5 mM glutamine. Cells were seeded at 0.4 million/ml and incubated in shaker flasks for 3 days. After 3-day cell culture, the cell pellets were collected and washed twice with phosphate buffered saline (PBS, pH 7.4) buffer. Cells were then lysed directly with 8 M urea/1 M NH_4_HCO_3_ solution and briefly sonicated until the solutions were clear. Protein concentrations were determined by bicinchoninic acid (BCA) protein assay reagent (Thermo Scientific, Fair Lawn, NJ, United States). Proteins were reduced by 10 mM DTT at 37°C for 1 h and then the cysteines on proteins were alkylated by 20 mM iodoacetamide for 45 min in dark. Then sequencing-grade trypsin (protein enzyme, 40:1, w/w; Promega, Madison, WI, United States) was added to the diluted proteins and incubated at 37°C overnight. The peptides were cleaned by C18 solid-phase extraction.

### 2.2 Glycopeptide Enrichment and Fractionation

The glycopeptides were enriched by mixed anion exchange (MAX) extraction cartridges and then fractionated by basic reversed-phase liquid chromatography (bRPLC) (Yang et al., 2018). In brief, 1 mg peptides were loaded into one Oasis MAX extraction cartridges (Waters) and about 200 µg glycopeptide eluate was collected together from three MAX cartridges. Then, the collected glycopeptides were desalted by C18 solid-phase extraction and injected into Zorbax Extend-C18 analytical column containing 1.8 μm particles at a flow rate of 0.2 ml/min. The glycopeptides were fractionated and collected into 96-well plates in a time-based mode from 16 to 112 min. The resulting 96 fractions of tryptic glycopeptide samples were further concentrated into 24 fractions by combining fractions 1, 25, 49, 73; 2, 26, 50, 74; etc. The 24 fraction samples were then dried in a speed-vac and stored at −80°C until LC-MS/MS analysis.

### 2.3 Nano LC−MS/MS Analysis

The IGPs were subjected to two LC-MS/MS analytical repeats per fraction in a Q-Exactive mass spectrometer (Thermo Fisher Scientific, Bremen, Germany). The samples were re-suspended with 3% ACN and 0.1% FA. 1 µg in 4 µl per sample was first separated in a Dionex Ultimate 3000 RSLC nano system (Thermo Scientific) with a PepMap RSLC C18 column (75 µm 50 cm, 2 µm, Thermo Scientific) protected by an Acclaim PepMap C18 column (100 µm 2 cm, 5 µm, Thermo Scientific). The mobile phase consisted of 0.1% FA and 3% ACN in water (A) and 0.1% FA, 90% ACN (B) using a gradient elution of 0–2% B, 1 min; 2–8% B, 9 min; 8–31% B, 80 min, 31–38% B, 20 min; 38–95% B, 5 min; 95% B, 10 min; 95–2% B, 4 min. The flow rate was kept at 0.3 μl/min. Data-dependent higher-energy collisional dissociation (HCD) fragmentation was performed on the 12 most abundant ions. The spray voltage was set to 1.5 kV. Spectra (AGC target 3  ×  10^6^ and maximum injection time 60 ms) were collected from 400–2,000  m/z at a resolution of 70, 000 followed by data-dependent HCD MS/MS (at a resolution of 35,000, NCE 32%, intensity threshold of 4.2  ×  10^4^, AGC target 2  ×  10^5^ and maximum injection time 120 ms) of the ions using an isolation window of 1.4  m/z. Charge state screening was enabled to reject singly charged ions and ions with more than eight charges. A dynamic exclusion time of 30 s was used to discriminate against previously selected ions.

### 2.4 MS Data Analysis

The MS data were searched by an in-house developed glycopeptide analysis software called GPQuest 2.0^20^. The database of glycosites was the glycosite-containing peptide data from our previous de-glycopeptide methods (Yang et al., 2018). The Cricetulus griseus glycan database was from a previous CHO cell glycomics profiling study (North et al., 2010). The databases contain 57,653 predicted glycosites and 343 glycan structure entries. Prior to database search, MSConvertGUI was used to convert the.RAW files to.mzML files with the ‘‘pick picking’’ option selected. The parameters for mass tolerance of MS1 and MS2 were 10 and 20 ppm, respectively. The spectra containing an oxonium ion *m/z* 204.09 were chosen for further searching. Results were filtered based on the following criteria: 1) false discovery rate (FDR) less than 1%, 2) ≥ 2 PSMs for each peptide were required, and 3) all the PSMs should be annotated by at least one N-linked glycans on one glycosites. All the core-fucosylated intact glycopeptides were verified by the peptide + GlcNAc2Fuc1 or peptide + GlcNAc1Fuc1 fragments in the MS/MS spectra.

### 2.5 Quantification of Glycoproteins and Intact Glycopeptides

For the glycoprotein quantitative analysis, the relative abundances of glycoproteins in each cell type were determined by the summed PSM value of each protein’s constituent glycopeptides from 24 fraction analyses in two replications together. This label-free quantification method was also applied for glycan quantitative analysis. The medium log2 ratio value of an identical glycoprotein or glycan was calculated by the ratio of the relative abundance of the identical glycoprotein or glycan in FUT8KO cells to that in WT cells. As in Figure 3B, the ratio of core-fucosylated glycoproteins in fucosylated glycoproteins was determined by their corresponding relative abundances for each glycoprotein.

### 2.6 Glycosylation-Related Enzymes and Their Glycosylation Analysis

The level of glyco-related enzymes, such as glycosyltransferases and glycosidases can influence the expression of some glycoproteins intracellularly and extracellularly. The identified glycoproteins were blasted with glycosylation-related genes data from RNA-seq analysis and classified into two major categories as glycosyltransferases and glycan degradation enyzmes and 24 sub-catergries like mannosyltransferases, galactosyltransferases and mannosidases, etc. (Xu et al., 2011). The illustration of glycosylated proteins was created using Tbtools (Chen et al., 2020), which relied on the relative abundance of each glycan composition at each glycosite. The schematic representation of the glycoprotein was generated from Pfam 34.0^24^.

## 3 Results and Discussion

### 3.1 Identification of Intact Glycopeptides in the WT and FUT8 KO CHO Cells

**FIGURE 1:**
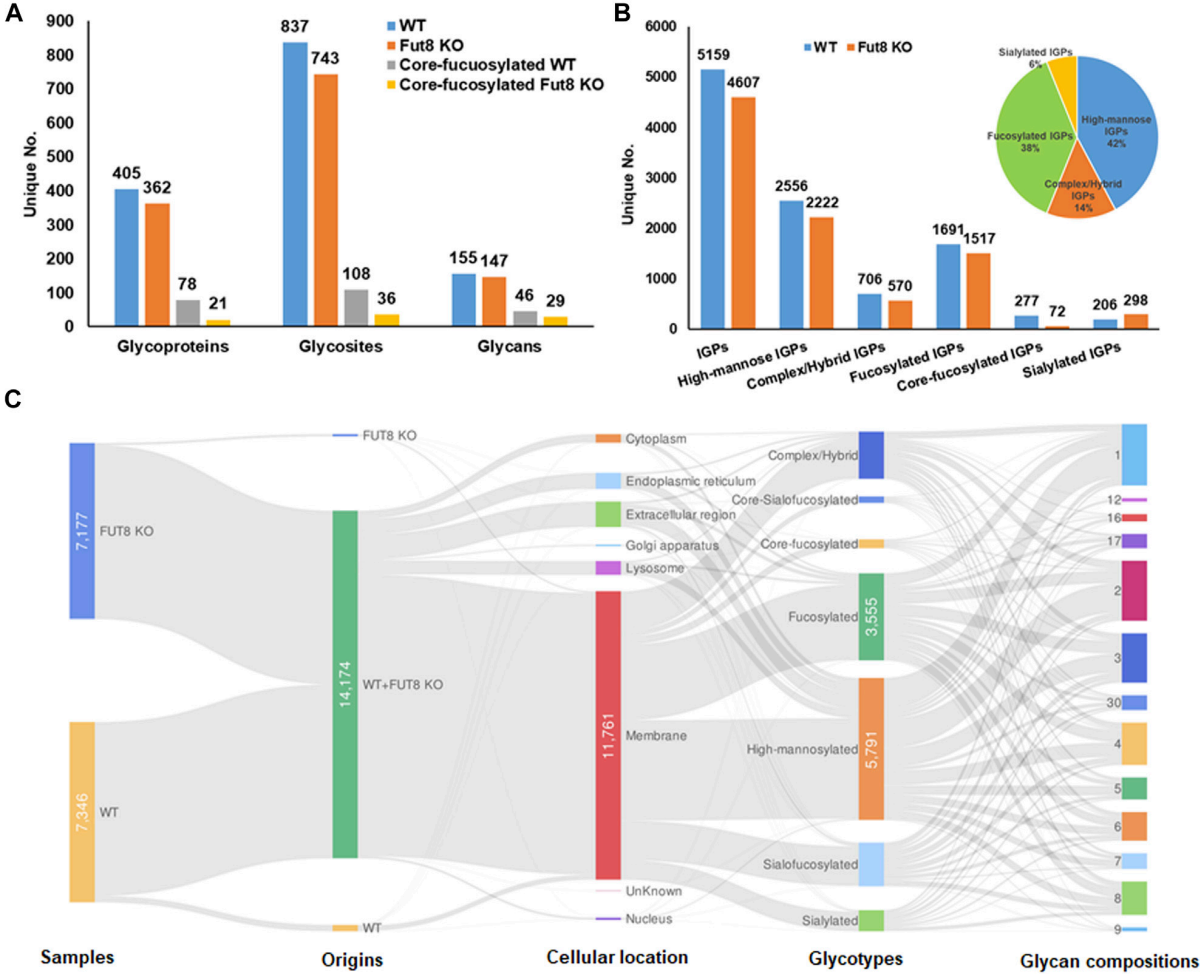
Identification and distribution of glycoproteins in wild type (WT) and *FUT8* Knock-out (KO) CHO-K1 cells. **(A)** Distribution of identified glycoproteins, glycosites and glycans in WT and FUT8KO CHO cells, **(B)** Identification of total and sub-glycotype IGPs identified from WT and FUT8KO CHO cells, showing that most IGPs are high-mannosylated both in WT and FUT8KO CHO-K1. **(C)** Illustration for distribution of protein glycotypes, cellular location and glycan compositions on the glycopeptides in WT and FUT8KO CHO cells.

To understand the role of FUT8 in the glycosylation of CHO cells, we developed a FUT8 knockout CHO cell line for large-scale glycoproteomic analysis (Wang et al., 2018). To characterize the glycoproteomics of FUT8KO as well as wild-type CHO cells, glycopeptides were enriched using hydrophilic MAX extraction column and fractionated by basic RPLC. The intact glycopeptides were analyzed by Q-Exactive mass spectrometer and identified by GPQuest 2.0. The assigned intact glycopeptides were filtered using peptide spectrum matches (PSMs) with a maximum of 1% false discovery rate (FDR), the morpheus score higher than 6, and the number of PSMs of peptide more than 2. A total of 25,859 intact glycopeptide spectra in WT CHO cells and 21,045 intact glycopeptide spectra in FUT8KO cells were annotated (Supplementary Tables S1, S2). In the WT CHO cells, 5,159 intact glycopeptides from 405 glycoproteins containing 837 glycosites and 155 glycan compositions were identified, while 4,607 intact glycopeptides from 362 glycoproteins, 743 glycosites, and 147 glycan compositions were identified in FUT8KO CHO cells. In combination, a total of 442 glycoproteins with 928 glycosites, 181 glycan compositions, and 7,127 unique N-linked glycosite-containing IGPs were identified from FUT8KO and WT CHO cells (Figure 1A). Interestingly, we noticed that the number of unique IGPs was decreased in the FUT8KO CHO cells. It showed the same tendency for the high-mannosylated, fucosylated and other complex or hybrid IGPs. Conversely, for sialylated IGPs, its number was increased in the FUT8KO cells (Figure 1B). In the WT parental CHO cells, approximately 5.36% in the total IGPs and 16.38% in all the fucosylated IGPs were core-fucosylated, which were confirmed by the peptide + GlcNAc2Fuc1 or peptide + GlcNAc1Fuc1 fragments in the MS/MS spectra. Meantime, there were about 1.56% in the total IGPs and 4.75% in all the fucosylated IGPs were core-fucosylated in the FUT8KO cells (Figure 1B). In the 7,127 IGPs identified from FUT8KO and WT cells together, about 95.92% IGPs were shared by both cell lines, indicating the genetic removal of FUT8 could rarely change the glycan composition of the protein glycosites. Moreover, about 42 and 38% glycosites in all glycosites were modified by high-mannosylated and fucosylated glycan structures, respectively (Figure 1B). We also noticed that 25.97% of the IGPs were super-microheterogeneity with six to ten glycan compositions per peptide sequence and 53.65% of the IGPs were hyper-microheterogeneity with more than ten glycan compositions at one glycosite. The average glycan compositions at one glycosites are about eight. From the cellular location distribution of the glycoproteins, it showed that most of the glycoproteins were membrane proteins (Figure 1C and Supplementary Table S3).

### 3.2 Relative Abundance of Glycopeptides in the WT and FUT8KO CHO Cells

**FIGURE 2:**
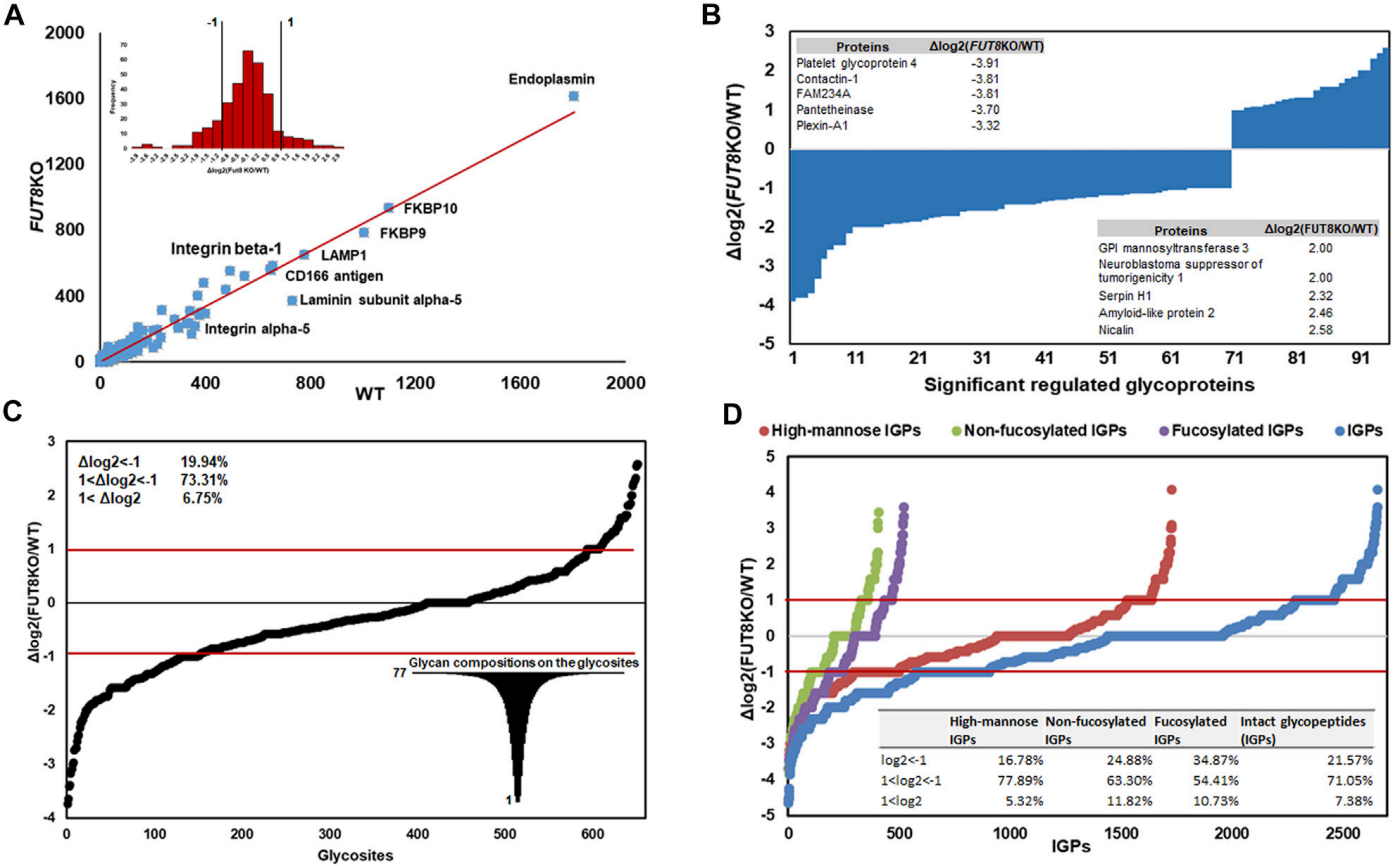
Quantitative analysis of glycoproteins in WT and FUT8KO CHO cells. **(A)** Relative abundance scatter and distribution profiling of glycoproteins between WT and FUT8KO CHO cells, showing a strong correlation between the WT and FUT8KO cells. **(B)** The ninety-five significant regulated glycoproteins between WT and FUT8KO cells, the top five up-regulated and down-regulated glycoproteins were also showed. **(C)** Differential glycosylation on the 652 glycosites between WT and FUT8KO cells. **(D)** Relative abundance distribution of sub-glycotypes of intact glycopeptides between WT and FUT8KO cells.

In principle, the FUT8 gene is involved in the core-fucosylation of glycoproteins, which catalyzes the transfer of a fucose residue to the innermost GlcNAc residue of N-linked oligosaccharide. To investigate the changing of the glycosylation regulated by FUT8, we performed a comparative analysis using label-free quantification based on spectral counting. To quantitatively analyze the glycoproteins, 325 glycoproteins identified in the replication of the two cell lines were analyzed as shown in Figure 2. It revealed that the protein Endoplasmin was the highest abundant glycoproteins in the CHO cells, which may participate in the unfolding of cytosolic leaderless cargos. From the relative abundance scatter in Figure 2A, it showed that there was no significant expression difference between the WT and FUT8KO CHO cells. The log2 (ratio) of 232 (71.38%) proteins were varied less than two folds. Meantime, 28.62% of the identified glycoproteins were significantly changed, in which 22 (6.77%) and 71 (21.85%) out of the 325 proteins were upregulated and downregulated in the FUT8KO CHO cells. The top five of the upregulated and downregulated proteins were showed in Figure 2B, which showed the downregulated proteins were more significantly changed than the upregulated proteins. Especially, the expression of platelet glycoprotein 4 was sharply decreased in the FUT8KO CHO cells compared to WT CHO cells, which was a multifunctional glycoprotein that acts as a receptor for a broad range of ligands. From the comparative analysis of the glycoprotein abundance in the WT and FUT8KO CHO cells, it indicated that FUT8KO CHO cells not only altered the glycan profile by intervening in the glycosylation process in *Golgi* but also regulated the relative abundance of some glycoproteins.

The number of N-glycans is a distinct feature of each glycoprotein sequence and cooperates with the physical properties of the *Golgi* N-glycan-branching pathway to regulate surface glycoprotein levels (Lau et al., 2007). To reveal the influence of FUT8 on the protein N-glycosylation, we also quantitatively analyzed the glycopeptides between the WT and FUT8KO CHO cells. It showed that the diversity of glycosylation was changed due to the knock-out of FUT8 in CHO cells. In this study, 928 glycosites were identified and 652 glycosites were identified both in the WT and FUT8KO CHO cells. In the 652 glycosites identified from two cells, approximately 478 (73.31%) glycosites were changed less than two folds, and 26.69% in the identified glycosites were significantly changed, in which 130 (19.94%) were downregulated and 44 (6.75%) were upregulated. The glycopeptides N_457_QT from CD166 antigen were most downregulated with the log2 (ratio) as −3.74 in the FUT8KO CHO cells (Figure 2C). Interestingly, we also noticed that a great diversity of N-glycosylation on these regulated glycosites. For example, there were 77 glycan compositions on N_67_SS from protein basigin.

In addition to the glycosites, the glycotype on IGPs was also altered in the FUT8KO CHO cells. Approximately 2,656 IGPs were identified both in the WT and FUT8KO CHO cells and about 71.05% of IGPs relative abundances were changed less than two folds. It indicated that fucosylated IGPs were altered significantly and about 238 out of 522 (45.60%) fucosylated glycopeptides were upregulated (34.87%) or downregulated (10.73%) in the FUT8KO CHO cells.

### 3.3 Differential Analysis of the Core-fucosylated Proteins in the FUT8KO and Wild-type CHO Cells

**FIGURE 3:**
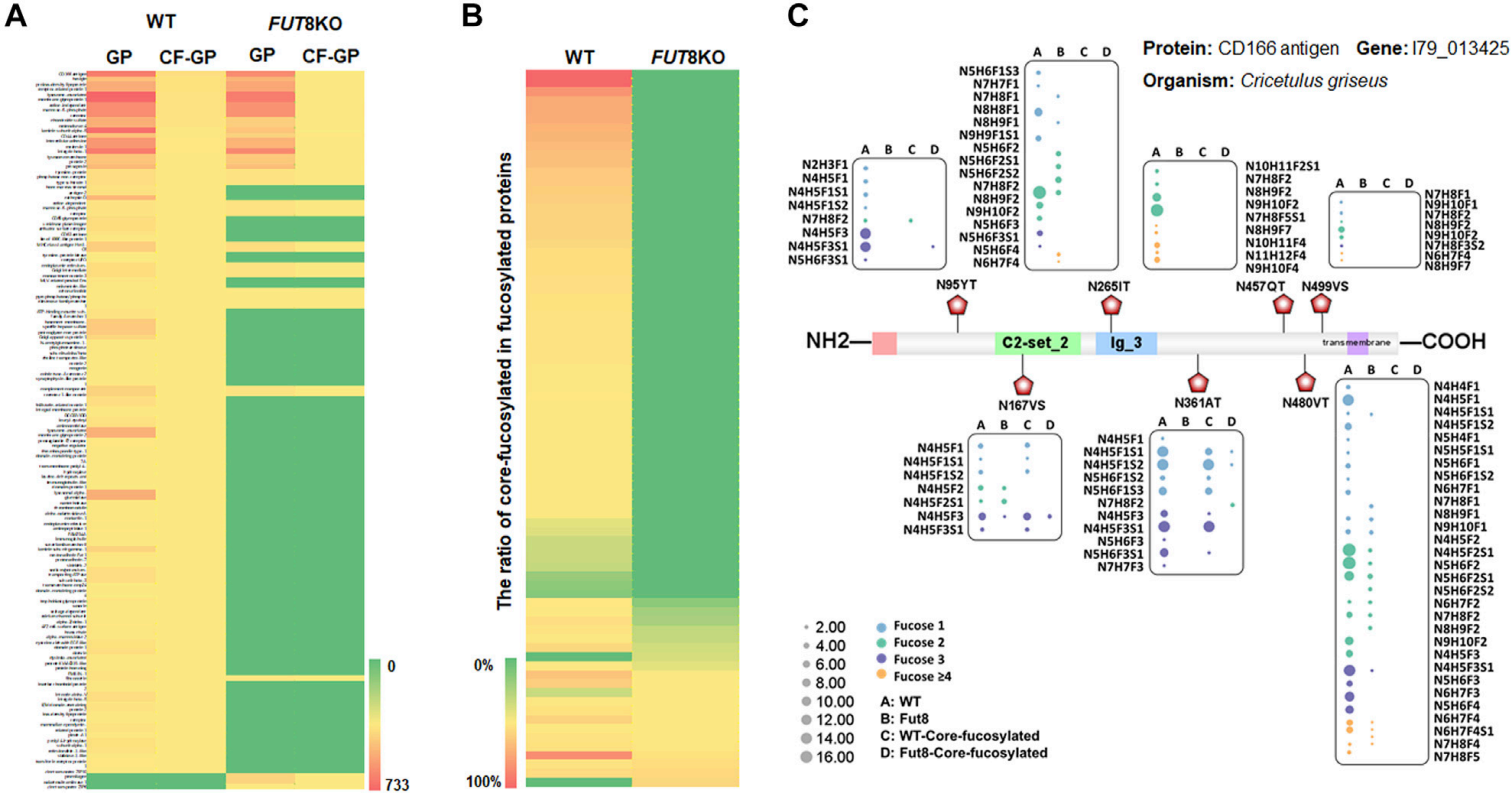
The change of fucosylated glycoproteins in the FUT8KO CHO cells. **(A)** Heatmap of fucosylated and core-fucosylated glycoproteins in the WT and FUT8KO CHO cells. **(B)** Percentage of core-fucosylated glycoproteins in fucosylated glycoproteins in the WT and FUT8KO CHO cells. **(C)** Fucosylation change of protein CD166 (7 glycosites) with the FUT8KO in the CHO cells.

From the relative abundance analysis of intact glycopeptides, we found that fucosylated glycopeptides changed significantly in FUT8KO CHO cells. A total of 309 core-fucosylated IGPs were identified, representing 114 glycosites, 52 glycans and 80 core-fucosylated proteins. 237 fucosylated IGPs from 59 (73.75%) glycoproteins totally lost their core-fucosylation in FUT8KO CHO cells. Moreover, 72 out of 4,607 IGPs from 21 glycoproteins were still identified as core-fucosylated glycopeptides with very low relative abundance in FUT8KO CHO cells. Besides the core-fucosylated proteins, the terminal-fucosylated proteins were also decreased in FUT8KO CHO cells (5 proteins significantly down-regulated and 21 proteins disappeared). Interestingly, 2 proteins (procollagen galactosyltransferase 1 and zinc transporter ZIP6) were not fucosylated in the WT but fucosylated in FUT8KO CHO cells (Figure 3A). It indicated that the ratio of core-fucosylated in all fucosylated was sharply decreased from 15.42% in WT cells to 1.14% in FUT8KO cells for glycoproteins and from 72.70% in WT cells to 16.18% in FUT8KO CHO cells for glycopeptides (Figure 3B). As one of the hyper-fucosylated glycoproteins, CD166 antigen has seven fucosylated glycosites identified in which three glycosites were core-fucosylated (Figure 3C). The most heterogeneous site N_480_VT of CD166 antigen contained 31 fucosylated glycan compositions. Moreover, the relative abundances of all the glycan types were decreased in FUT8KO CHO cells. The core-fucosylated glycosylation of the three glycosites on CD166 antigen were almost disappear in the FUT8KO CHO cells (Figure 3C).

### 3.4 Alteration of the N-Linked Glycans and Glycosylation-Related Enzymes in FUT8KO CHO Cells

**FIGURE 4:**
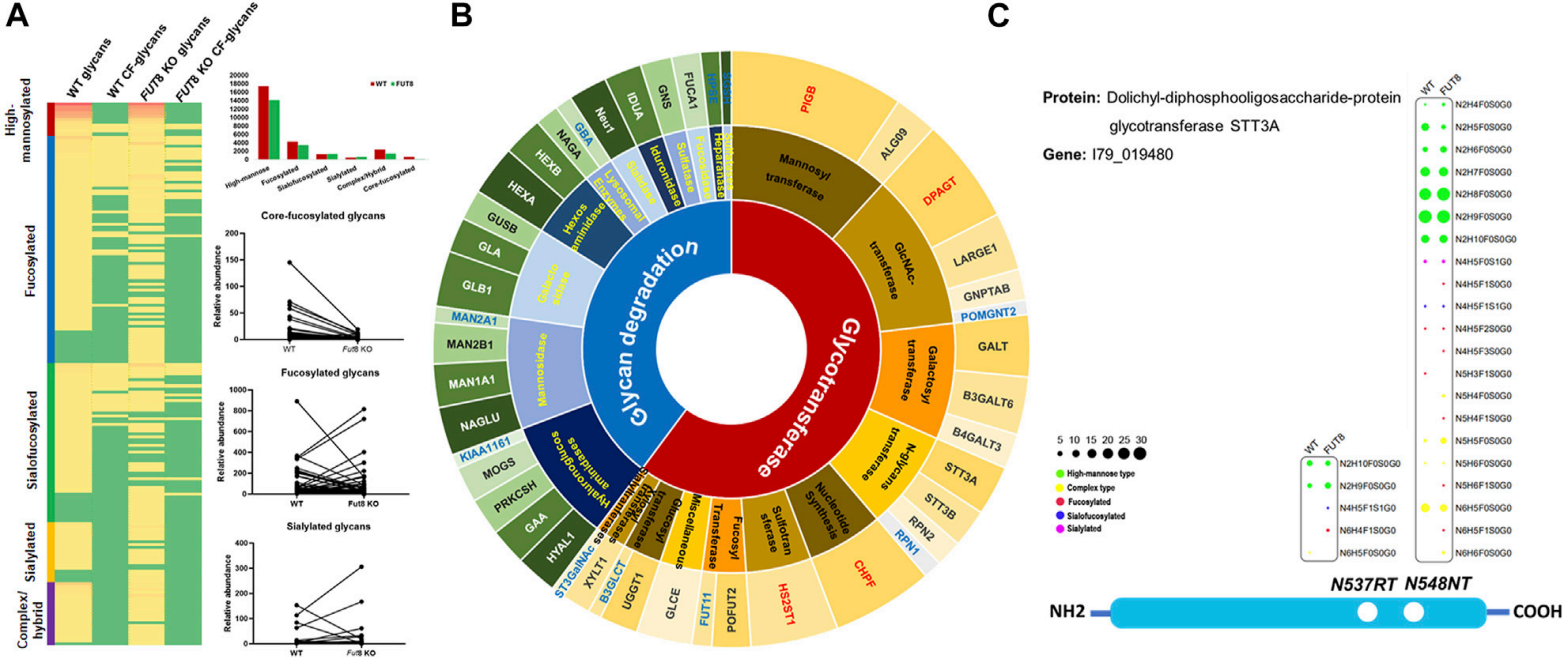
The change of glycosylation and expression of glycosylation related enzymes in FUT8KO CHO cells. **(A)** The change of glycans on the glycoproteins in the WT and FUT8KO CHO cells. **(B)** The expression of glyco-related enzyme in the WT and FUT8KO CHO cells. **(C)** The glycosylation of N-glycan transferase dolichyl-diphosphooligosaccharide-protein glycosyltransferase subunit STT3A.

Furthermore, to investigate the influence of FUT8KO on the N-linked glycans, the glycans from IGPs were analyzed and 181 glycans were identified in total and grouped into five subtypes, high-mannose, fucosylated, sialofucosylated, sialylated and other complex or hybrid N-glycans. About 34 and 26 glycans were only presented in the WT and FUT8KO cells. The relative abundances of 36 glycan compositions were increased and 46 glycan compositions were decreased more than two folds in the FUT8KO CHO cells. From the quantitative analysis of glycan subtypes in Figure 4A, it displayed that the core-fucosylation nearly disappeared. The high-mannosylated and fucosylated glycans were generally decreased while sialylated glycans were increased.

The glycosylation of protein is a complex and multistep networking that employs about 200 glycotransferases and glycosidases. These enzymes determine which protein could be glycosylated, the glycosylation site of glycans on the proteins and the glycan structures on the sites. Studies of deficiencies in glycosylation-related enzymes could be facilitated to understand the biological functions of protein glycosylation. From our high-throughput site-specific glycoproteomics, it showed that around 51 glycosylation-related enzymes were also glycosylated, which were classified into 28 glycosyltransferases and 23 glycosidases. It has been reported that 159 glycosylation-related genes were expressed in the CHO cell by RNA-seq data (Xu et al., 2011). It also showed that the relative abundance of 4 glycosyltransferases was obviously increased and 12 glycosylation-related enzymes were obviously decreased in the FUT8KO CHO cells, and all the significantly changed glycan degradation enzymes were decreased (Figure 4B and Supplementary Table S4). In these glycosylation-related enzymes, lysosomal alpha-glucosidase was the most abundant proteins and with the greatest macroheterogeneity (6 glycosites) and microheterogeneity (17 glycan compositions on N_134_LS). Dolichyl-diphosphooligosaccharide-protein glycosyltransferase subunit STT3A catalyzes the initial transfer of Glc_3_Man_9_GlcNAc2 from the lipid carrier dolichol-pyrophosphate to an asparagine residue in nascent polypeptide chains in the endoplasmic reticulum. It is present in the majority of oligosaccharyltransferase (OST) complexes and mediates N-glycosylation of most sites on target proteins. Three glycosites were identified from the human liver tissue (Chen et al., 2009) and two glycosites were identified from CHO cells either by the de-glycosylated glycopeptide or site-specific intact glycopeptide identification methods in our previous study (Yang et al., 2018). In this study, the two glycosites were identified with 21 glycan compositions on N_544_NT and 5 glycan compositions on N_537_RT. Interestingly, the glycan compositions on N_544_NT from STT3A were increased in the FUT8KO cells, which were modified with 16 glycan compositions in WT and 20 glycan compositions in FUT8KO CHO cells (Figure 4C).

### 3.5 Discussion

In our previous study, we developed a one-step glycopeptide enrichment method and applied this methodology to analyze the protein glycosylation of FUT8KO cells as a proof of concept, from which we identified 2,634 unique IGPs from 243 glycoproteins with 459 glycosites (Yang et al., 2020). Some interesting results indicated that the core-fucosylated glycopeptide was still present in the FUT8KO CHO cells. To comprehensively decipher the protein glycosylation in the FUT8KO CHO cells, we performed a thorough analysis of the site-specific protein glycosylation of the FUT8KO CHO cells using the comprehensive glycoproteome method in this study. 7,127 unique IGPs from 442 glycoproteins with 928 glycosites were identified, which showed more than two-fold IGPs and glycosites identification compared to our previous study (Yang et al., 2020).

To explore the role of core-fucosylation in cells, it is desirable to comprehensively analyze the site-specific fucosylated glycoproteins. In this study, 72 core-fucosylated glycopeptides out of 4,607 IGPs were identified from FUT8KO CHO cells which are from 21 glycoproteins with very low core-fucosylation. The average ratio of core-fucosylated proteins in the glycoproteins was decreased from 0.13 in WT to 0.03 in FUT8KO CHO cells. For these 21 core-fucosylated glycoproteins, most of them were distributed on the plasma membrane and lysosomal membrane. It indicated that the core-fucosylation of these proteins was related to receptor activity, cell adhesion and migration.

As a non-template-mediated post-translational modification (PTM), glycans are not directly regulated by the genetic code. Instead, the glycosylation of proteins was controlled by the expression levels of glycosyltransferase and glycosidase enzymes. The observation that the N-glycan abundance is decreased in the FUT8KO CHO cells compared with WT CHO cells suggests that there is dysregulation of other enzymes responsible for N-glycan biosynthesis. OST complex catalyzes the transfer of the oligosaccharide from Dol-P-P to Asn-X-Ser/Thr in newly synthesized regions of proteins (Stanley et al., 2017). It is composed of couples of subunits contained STT3A/STT3B, OST4, RPN1, RPN2, etc. The RPN1 and RPN2 were considered to provide binding sites for translating ribosomes, associate with translocon and enable co-translocational glycosylation of entering polypeptides (Mohorko et al., 2011). The significant down-regulation of OST complex subunit RPN1 and RPN2 depressed the expression of all N-linked glycoproteins and the glycosylation level of the glycoproteins. Hence, targeting biosynthetic steps in glycosylation generally results in predictable global outcomes, but can also causes some minor unpredictable glycosylation effects. Therefore, it is desirable to monitor the glycosylation of the proteins in glycoengineered cells.

In this study, we performed an integrated and comprehensive glycoproteomic analysis of FUT8KO and parental wild-type CHO-K1 cells and compared the site-specific protein glycosylation and glyco-related enzymes difference between these two cell lines. By the site-specific glycosylation analysis method, we investigated the influence of FUT8 gene knock-out on proteins glycosylation and their site specificity. Moreover, the whole glycosylation biosynthetic pathways of CHO cells were also changed in the FUT8KO CHO cells. The glycoproteomic data will improve our understanding of the roles of α1,6-fucosyltransferase and the resulting core-fucosylation in CHO cells.

## Data Availability

All datasets presented in this study are included in the Supplementary Tables S1 and S2.

## Abbreviations

IGP, intact glycopeptide; CHO, Chinese hamster ovary; PSM, peptide spectrum match; LC-MS, liquid chromatography mass spectrometry.
